# Efficient automated error detection in medical data using deep-learning and label-clustering

**DOI:** 10.1038/s41598-023-45946-y

**Published:** 2023-11-09

**Authors:** T. V. Nguyen, S. M. Diakiw, M. D. VerMilyea, A. W. Dinsmore, M. Perugini, D. Perugini, J. M. M. Hall

**Affiliations:** 1 Presagen, Adelaide, SA 5000 Australia; 2 School of Computing and Information Technology, University of Wollongong, Wollongong, NSW 2522 Australia; 3 Ovation Fertility, Austin, TX 78731 USA; 4 Texas Fertility Center, Austin, TX 78731 USA; 5 California Fertility Partners, Los Angeles, CA 90025 USA; 6 Adelaide Medical School, The University of Adelaide, Adelaide, SA 5000 Australia; 7 Australian Research Council Centre of Excellence for Nanoscale BioPhotonics, Adelaide, SA 5005 Australia; 8 School of Physical Sciences, The University of Adelaide, Adelaide, SA 5005 Australia

**Keywords:** Health care, Medical research, Mathematics and computing

## Abstract

Medical datasets inherently contain errors from subjective or inaccurate test results, or from confounding biological complexities. It is difficult for medical experts to detect these elusive errors manually, due to lack of contextual information, limiting data privacy regulations, and the sheer scale of data to be reviewed. Current methods for training robust artificial intelligence (AI) models on data containing mislabeled examples generally fall into one of several categories—attempting to improve the robustness of the model architecture, the regularization techniques used, the loss function used during training, or selecting a subset of data that contains cleaner labels. This last category requires the ability to efficiently detect errors either prior to or during training, either relabeling them or removing them completely. More recent progress in error detection has focused on using multi-network learning to minimize deleterious effects of errors on training, however, using many neural networks to reach a consensus on which data should be removed can be computationally intensive and inefficient. In this work, a deep-learning based algorithm was used in conjunction with a label-clustering approach to automate error detection. For dataset with synthetic label flips added, these errors were identified with an accuracy of up to 85%, while requiring up to 93% less computing resources to complete compared to a previous model consensus approach developed previously. The resulting trained AI models exhibited greater training stability and up to a 45% improvement in accuracy, from 69 to over 99% compared to the consensus approach, at least 10% improvement on using noise-robust loss functions in a binary classification problem, and a 51% improvement for multi-class classification. These results indicate that practical, automated a priori detection of errors in medical data is possible, without human oversight.

## Introduction

Medical datasets can often contain inherent errors due to uncertain diagnoses, potential subjectivity in medical practitioners’ interpretation of test results, or confounding variables stemming from underlying biological complexities in the patient. Results of medical tests therefore represent measurements that do not always perfectly match the ground truth of a diagnosis or condition of a patient. Such errors in ground truth outcomes represent a significant challenge for the development of artificial intelligence (AI) for healthcare applications. The presence of errors in medical datasets can severely hamper the reliability and performance of these AI algorithms^1,2^, potentially placing patients at risk^3^. Good quality, error-free data are also critical for emerging applications such as precision medicine^4^, as well as clinical trial data used to support approvals for new patient treatments^5^.

Identifying errors in datasets is not straightforward. First, manual identification of errors can be haphazard and lack scalability. Secondly, datasets needed to train AI models in many industries are already at a size where manual data verification is not feasible^6^. In industries such as healthcare, privacy policies or laws may prevent third parties conducting manual verification of data^1,7^. Sources of mislabeling that rely on the judgment of medical professionals or that contain a certain element of subjectivity and interpretation, such as medical test outcomes or diagnoses, cannot always be manually identified.

One key insight drawn from the application of deep learning for identifying poor quality data is that issues with quality can fall into different categories^1,8^: (a) *mislabeled* data, where features in the data are clearly identifiable, but they are simply incorrectly labeled (i.e. data errors), and (b) *noisy* data, where the data are unclear or ambiguous, lacking features to make definitive conclusions regarding their ground truth outcomes, which is often called feature noise^9,10^. These two types of factors, when present in a dataset, can both negatively impact AI performance and reliability^1,8^. Being able to identify where a given datapoint in a dataset lies on a spectrum of label-noise is therefore of paramount utility in creating reliable AI.

In this work, a novel deep learning method for automatic a priori identification of data errors is presented. It is intended to be integrated into the training process for AI models, and extends the Untrainable Data Cleansing (UDC)^1^ technique with a label-clustering algorithm. This novel algorithm, which we denote the LDC, generates a continuous label-noise confidence score for a given dataset, which can then be used to identify the likelihood that each datapoint is either correct, or erroneous (mislabeled) or noisy (unclear). A high score for a datapoint indicates a high degree of confidence that the datapoint is mislabeled. A low score indicates that the datapoint is likely correctly labeled. A medium score indicates that is not a high confidence in the label either way, and that the datapoint is noisy.

If the ground truth label is known, then the performance of the LDC algorithm in identifying errors in the data can be measured using the Intersection over Union (IoU) score or the error identification accuracy, defined in "Experiment design" section The IoU score measures how well two subsets of data overlap. If all the errors in the data are correctly identified, and no data are identified as errors that are not actually errors, then IoU will take a value of 1.0. If some errors go undetected, the IoU value will drop. Similarly, if data are wrongly identified as errors, the IoU value will also drop, to a minimum value of 0.0. Thus, the IoU score penalizes failure to identify errors, but also wrongly identifying errors in correct data.

If the ground truth label is not known or is uncertain, then the performance of the LDC algorithm can be assessed using ML model performance improvement after LDC cleansing of training data. The LDC was tested on a range of diverse datasets from different fields of physiology, including human activity recognition (HAR) time-series data^11^, patient treatment records^12^, and human oocyte images^13^.

Measurement of the performance of the LDC is achieved by training an AI model on the original (poor quality) training set, then training on the LDC cleansed training set, and then comparing the two on a cleansed validation set. Firstly, training on cleansed dataset would typically be more stable (reduced oscillating loss function from epoch to epoch due to mislabeled data). Secondly, testing the AI model on a cleansed validation set shows that the cleansing has allowed a consistent and reliable model to be trained, in the absence of mislabeled or confusing data.

Our results showed a high level of accuracy in detecting poor quality data, and dramatic improvements to ML model performance when trained on LDC cleansed datasets. Results indicated that a deep learning label-clustering approach is an efficient method for automating data cleansing for both record and image data, allowing for the subsequent creation of increasingly reliable and scalable AI.

The novel contributions of this work are summarized as:

- The introduction of an efficient and robust data cleansing method (LDC) for identifying errors in datasets, especially in situations where manual methods are impractical or impossible. We introduced a novel metric-based method for effectively measuring the ability of the LDC to identify errors and noisy labels;
- Demonstration of the LDC across multiple types of data—time-series, record-based and image-based medical datasets where experimental results showed an uplift in AI performance due to the application of LDC. We introduced Histogram-based heuristic methods and visualizations for identifying subsections of the dataset that may be mislabeled, or simply noisy data (or ‘hard’ cases).

This article is organized as follows. Related works are summarized in "Related works" section. Methods are described in "Methods" section, including the experimental design in "Experiment design" section, label-noise confidence algorithm in "Label-noise confidence algorithm" section, and the composition of the non-medical and medical datasets in Sections "Dataset composition and settings" section. Results are presented in "Results" section, divided into sections by topic: a model consensus approach is compared to the new label-clustering method for a HAR test case in "Comparing label-clustering with different approaches for human activity recognition" section, including a comparison with existing loss functions robust to label noise. The LDC method is then applied to HAR and medical records for Patient Care in "Application of LDC to HAR and patient care" section. Medical images for denuded human oocytes are examined in "Application of LDC to labeled oocyte images" section. The Discussion is presented in "Discussion" section.

### Related works

Recent developments in the field of machine learning (ML) for cleansing poor quality datasets fall into several categories: (a) use of an updated model architecture that is robust to sources of feature noise while simultaneously not overfitting training dataset^8,9^, (b) more sophisticated regularization techniques such as dropout, batch-norm, augmentation and weight decay, which can partially improve model robustness to label noise^10^, (c) robust loss functions used during training, and d) selecting a subset of data that contains cleaner labels, which typically involves some kind of Hybrid learning, such as multi-network or multi-round learning, together with loss reweighting^9^.

Improvements in neural network architectures to maintain robustness to label noise during training typically involve the introduction of new layers with specific functionality, which either attempt to estimate the true label during training, such as NLNN^14^ or label-flip modeling^15^, or otherwise reduce the effects of label noise on training such as dropout regularization^16^. One method for reducing the effect of label noise is *mixup*^17^*,* which synthesizes new training data by combining pairs of datapoints and their labels with a particular distribution, and is purported to be advantageous due to the increased difficulty in overtraining on the interpolations between incorrect labels compared to those of the correct labels^8^.

In this work, dropout, batch-norm, augmentation and weight decay approaches will be used in all experiments to maintain model robustness, while focusing on data cleansing predominantly.

In improving the loss function, unbiased estimators can be introduced into the training process, which modify the loss function to be more robust^18^. While this can purportedly help to minimize the risk of random classification noise in a dataset, it does not directly address the deleterious effects caused by mislabeling, working well only in simple cases, and for a limited number of classes in classification problems^9^. A different approach involves adding a ‘label noise model’ into a deep learning framework, which can output a log-likelihood value^19^. This method presupposes a portion of the dataset can reliably be identified as noise, requiring prior annotation, and thus limiting its applicability outside its original domain. More recent methods have adopted a Bayesian framework^20^, where a Gaussian-shaped *prior* over the weights is added, minimizing this new loss function to capture a new ‘noise’ parameter in the outputs of the model (rather than in the data). The inferred noise is associated with inherent randomness of the data, and excludes the effects of systematic mislabeling stemming from confounding factors. Robust loss functions are explored in Comparing with a robust loss function approach to binary or multi-class classification" section as a comparator to data cleansing, exhibiting a more limited benefit especially in the case of binary classification.

In data cleansing, many traditional methods can be used to identify and remove, or reclassify data with suspected mislabeling, such as bagging, boosting, *k*-Nearest Neighbor and other forms of outlier detection^9^. More sophisticated hybrid methods have been proposed, such as iterative forms of self-learning, multi-round learning^21^, collaborative co-training^22^, or selection of representative data during each epoch, performing a ‘label correction’ step prior to starting the next epoch^23^. While these method allow for real-time detection of noise, they do not necessarily provide transparency as to which data are noisy or mislabeled, and the degree of confidence in the classification, and therefore it is not as useful for medical applications where these considerations are paramount.

The focus of this present work is on data cleansing. While the diverse array of methods described above can be used in complement, the advantages of data cleansing is that it not only can assist with explainability, triaging flagged data for further analysis for identification of possible sources of mislabeling and confounding factors leading to new insights on the dataset composition, it also assists in identifying subsets of the data on which an AI model might be expected to work best. This is especially important for medical datasets, where mislabeling can occur due to unclear diagnoses, subjectivity of medical test results, or patient medical indications, which may not be explicitly apparent in the dataset itself.

This work builds on a previously developed process that uses *k*-fold cross validation to obtain an aggregate of the model predictions^1^. This method is novel in that it can robustly detect noise by using multiple, contrasting neural networks (different initial conditions and architectures) to carry out a consensus approach for each datapoint, rather than being constrained to rely on the inference predictions of a single model. This ameliorates one of the main weaknesses of data cleansing, in that over-cleansing (removal of too many correctly-labeled datapoints) is reduced, as measured by IoU score. Additionally, the computational inefficiency is drastically reduced by entering scores obtained from one or more ML models into a label-clustering algorithm to obtain new label-noise confidence scores that can be determined without knowledge of the quality of each datapoint in advance.

## Methods

### Experiment design

The two types of problems, time-series and/or record-based datasets, and image-based datasets, require different model architectures. In each experiment two datasets are defined: the training/validation set, ${\mathbb{D}}$, (which are treated together using the *k-*fold cross validation method) and a holdback set. Additionally, for all experiments, model hyperparameters were tuned and selected by running multiple model training rounds with different sets of hyperparameters using the training set, and assessing model performance on a validation set. The ratio of training and validation set size is 80:20, using 5 folds for the cross validation. The results are then reported on the final holdback set.

To distinguish cleansed and uncleansed reporting sets, if the holdback set is a blind set to which LDC was not applied, it is called the test set. Otherwise, it is called a validation set.

For both problem types, log-loss was utilized to assess the best AI model performance while the AI models were reported using total accuracy (total number of correctly predicted datapoints across both classes), specificity, and sensitivity metrics, unless otherwise stated.

#### Intersection over Union score

The Intersection over Union (IoU) score represents one option for the assessment of the performance of error identification using UDC or LDC methods. IoU score is defined in terms of the total number of datapoints *correctly* identified as errors, $${N}_{correct}$$, known as the ‘intersection’ of prediction and ground truth, and the total number of datapoints that were either actual errors or predicted as errors $${N}_{actual or predicted}$$, known as the ‘union’:

1 $$IoU = \frac{{N_{correct} }}{{N_{actual\, or \,predicted} }}$$

If the error identification method identifies all actual errors within the data, and simultaneously does not identify any *correct* data as being errors, then the intersection $${N}_{correct}$$ will exactly equal the union $${N}_{actual or predicted}$$, that is, the algorithm only made predictions equal in number to the total actual errors in the data, and the IoU score is equal to 1.

#### Error identification accuracy

The error identification accuracy (EIA) can also be used for the assessment of error identification performance, and leads to a cleaner definition of cleansing performance for AI training, where removal of superfluous clean data is not problematic. This is because the EIA value does not penalize the removal of correct data, unlike the IoU. This is a useful accuracy measure for ML methods applied to Big Data, because the removal of clean datapoints typically only deteriorates model training if the dataset size is near a minimum threshold size.

EIA is defined as follows:

2 $$EIA = \frac{{N_{correct} }}{{N_{predicted} }}$$

where ${N}_{predicted}$ is the number of predictions made by the error identification method.

#### Deep learning architectures

For record-based experiments, the model was defined as having several 1D-CNN layers^24^, followed by a dropout layer for regularization. Then, the output features were flattened to a one-dimensional vector and passed through fully connected layers before reaching the output layer, which was used for predictions. The number of 1D-CNN and fully connected layers along with the feature maps and kernel sizes were made configurable and fine-tuned along with model architecture and conventional ML hyperparameters. Multi-layer perceptron (MLP) models^25^ were also used and compared to the performance of the 1D-CNN models, but consistently showed inferior results. The 1D-CNN model was sufficient for representing the clean HAR dataset without overfitting, prior to testing the effectiveness of the data cleansing method.

For 1D-CNN models, the Rectified Adam (RAdam)^26^ version of stochastic gradient descent was used for network optimization.

For image-based experiments, detector and segmentation models were used for image pre-processing, with architectures and parameters defined below. Binary classification models were used based on the pre-processed image inputs, with architectures and parameters also defined below.

All deep learning runs in this work make use of data normalization as a pre-processing step, and dropout layers to improve robustness to label noise. Further regularization steps in image processing are described below.

##### Detector model

A Faster R-CNN^27^ model was used. It included a ResNet50 feature pyramid network backbone^28^, and expects as input an image that has been scaled so that the height and width are both 800 pixels, to keep the resolution consistent for object detection. The output of the model was a bounding box corresponding to the detected object. A non-maximum suppression algorithm was also used, with a threshold value set to 0.25, and a score threshold set to 0.4.

The minimum box heigh and width admitted was 30 pixels. Small, detected boxes (with at least one box edge smaller than 30 pixels) that lie completely within another box were removed to prevent the model from under-fitting the boundary. In cases where two over-lapping boxes were output, the larger box encompassing the two boxes was selected.

##### Segmentation model

A U-Net architecture^29^ with a ResNet-50 encoder^27^ was trained to detect the *zona pellucida* region from 1180 pre-annotated oocyte images from California Fertility Partners, with a segmentation threshold of 0.7 and a segmentation width and height of 250 pixels each. The uncleansed validation set comprised 236 images, with performance of the final model evaluated using an image IoU score of > 95%.

##### Classification models

The deep learning architectures utilized for image classification included ResNet-34, ResNet-50^28^, Wide_ResNet-50_2^30^, DenseNet-121 and DenseNet-161^31^ all of which were pre-trained by using ImageNet^32^ and run for 100 epochs.

Learning rates ranged from 1.0e-4 to 3.0e-4 using Cosine Annealing as the scheduling technique, momentum values from 0.9 to 0.95, a dropout rate of 0.10 to 0.15, batch sizes of 8 to 32, and uniform RGB normalization. Stochastic gradient descent (SGD)^33^ was used for network optimization while categorical cross entropy was the loss function throughout. The *k-*fold cross validation was used in identifying the model consensus in UDC, while it was used to promote the training data randomization and varying the evaluation conditions for LDC, for *k* = 5.

All image classification training runs make use of batch-norm^34^, weight decay and heavy augmentation (cropping, scaling, rotation, color rotation, blurring and coarse-dropout) as further regularization techniques.

These parameters were used for all three types of image segmentation defined in "Application of LDC to labeled oocyte images"section.

### Label-noise confidence algorithm

**Figure 1:**
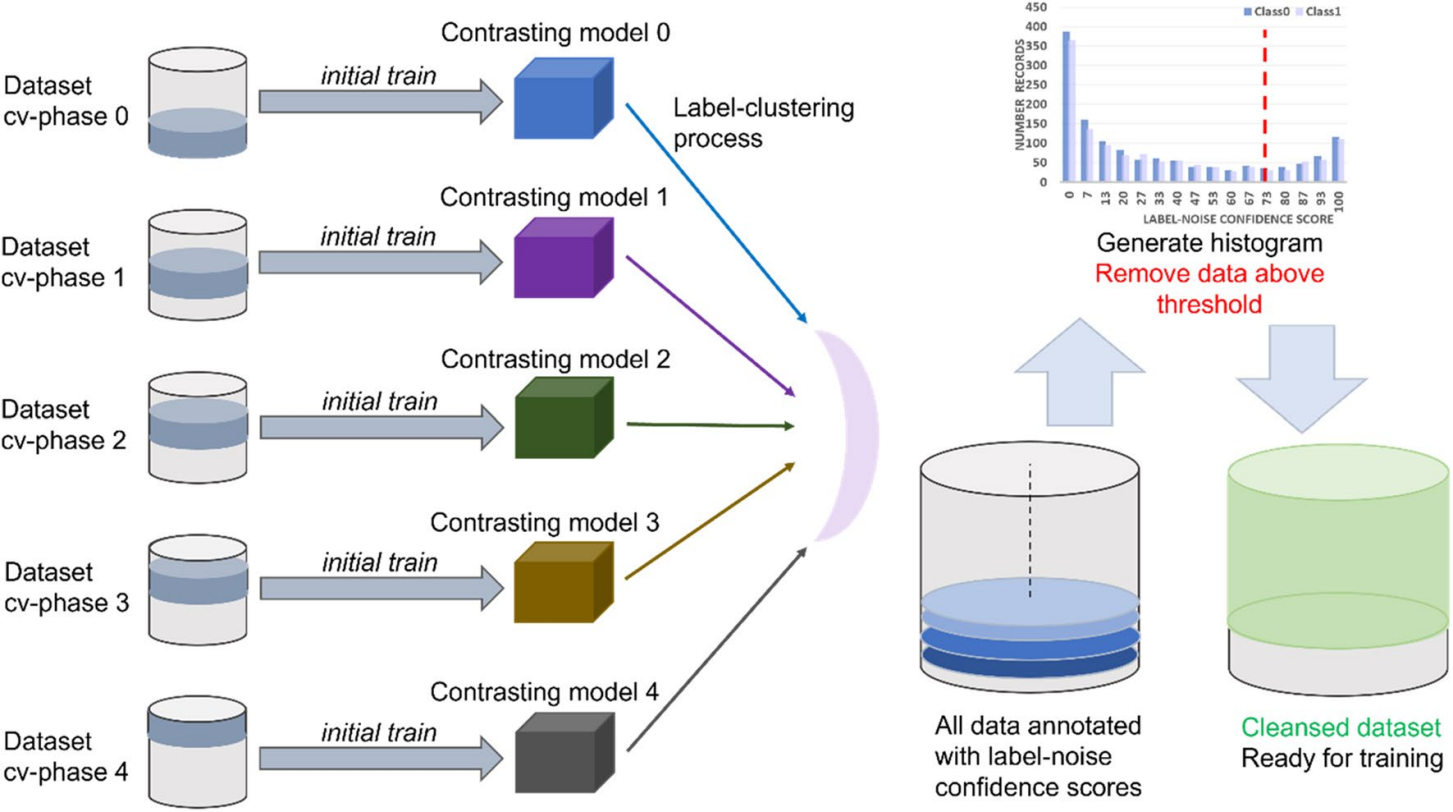
The workflow for the LDC method is illustrated, for both binary and multi-class classification problems. The process for a model single architecture and hyperparameter set is shown. This process involving *k-*fold cross validation may be repeated multiple times with different model settings and all such models may contribute evenly to the generated histogram as shown.

Figure 1 shows a visual representation of the steps LDC uses for identifying errors. Contrasting model configurations are trained on a given dataset, which can be achieved by changing the architecture and hyperparameters, or by performing a *k*-fold cross validation (as pictured) or both. Then, from the model inferences at each datapoint, the label-clustering algorithm is applied, described below. The distances from the center of gravities are normalized so that each datapoint has a corresponding label-noise confidence score. The scores may be binned into a histogram that shows the distribution of noise and mislabeling in the dataset, and threshold may be set for removal of errors prior to training a final model.

The reasoning behind these choices in constituent models to the LDC method is that, typically, any one ML model trained on a dataset will contain inherent bias, exhibited by a preference in classifying one subset of the data (in some cases, a one whole class) better than another due to small differences in the clarity of the features learned from the dataset. In order to mitigate the inherent bias in any one particular model, different folds of the data are used for the validation set, and different hyperparameters are selected, so that the consensus of multiple, contrasting models is used to drive error identification. This is a central point, that each model must be trained on a different subsection (i.e. cross-validation phase) of the training set, using the rest as its validation set, and each model may have different architectures, hyperparameters and inherent biases, thus making a consensus among the models on the predicted label of a datapoint more meaningful. If the models were all trained on the same cross-validation phase and the same settings, their concordances would likely be very high for all datapoints and so would not add any new information.

While training large numbers of neural networks is time-consuming and expensive, LDC can be customized so that only a few, high-performing model configurations are needed to generate a continuous label-noise confidence score.

Label-noise confidence scores defined the likelihood that an input sample was incorrectly labeled, or identified as too uninformative to be labeled as any particular class. These scores were used to identify poor quality data as part of this algorithm.

Note that the use of various model architectures and *k*-fold cross validation in the LDC algorithm, Algorithm 1, helped to introduce variations with respect to network structures and training data randomization, respectively. The rotation of validation sets into *k* folds increased the diversity of the initial conditions of the models considered. A histogram of ${\overline{l} }_{j}$ values (with increasing ${\overline{l} }_{j}$ on the x-axis), shows data sample distribution over different label-noise confidence scores.


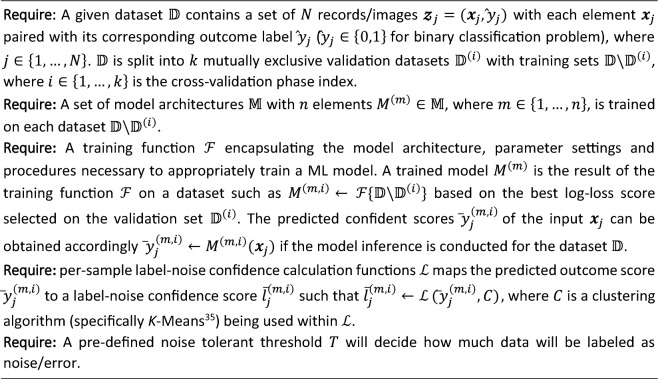
 
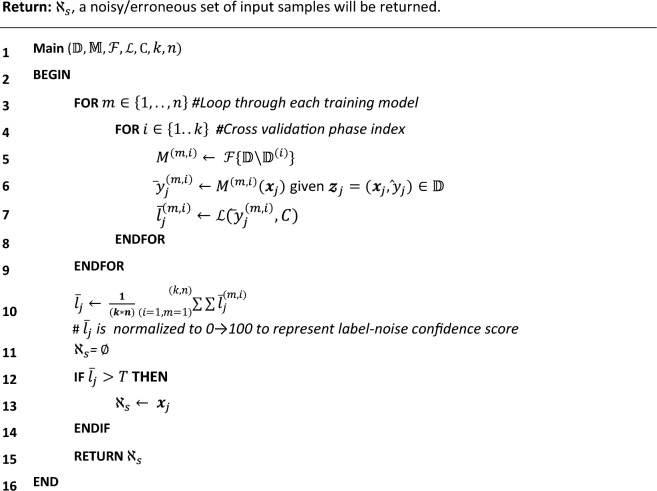


Algorithm 1 can be interpreted as follows. For each model architecture and associated hyperparameter set, the $k$-fold cross validation is deployed (line 4). Thus, each model architecture is utilized repetitively $k$-phase times, and for each phase, the model is trained with a different training dataset ${\mathbb{D}}\backslash {\mathbb{D}}^{\left(i\right)}$ and calibrated by the validation set ${\mathbb{D}}^{\left(i\right)}$. After training, the best model ${M}^{\left(m,i\right)}$ associated with the cross-validation phase $i$ and model architecture $m$ is obtained (line 5). The best epoch selection procedure involves finding the best value of a given evaluation metric on the validation set. Unless otherwise mentioned, log loss is selected as the key evaluation metric. It is important that the validation dataset is included in the cleansing process, as it is needed to effectively select a good candidate model prior to reporting on a further, holdback test set.

A prediction confidence score ${\overline{y} }_{j}^{\left(m,i\right)}$ of an input sample ${{\varvec{x}}}_{j}$ is calculated (at line 6), by running ${M}^{\left(m,i\right)}$ of the evaluation mode. The model inference is made against the entire dataset ${\mathbb{D}}$. ${\overline{y} }_{j}^{\left(m,i\right)}$ would represent how likely is ${{\varvec{x}}}_{j}$ belonged to class 0 or to class 1 (e.g. for the case of a binary classification problem).

Then, a label-noise confidence score ${\overline{l} }_{j}^{\left(m,i\right)}$ is computed for each ${{\varvec{x}}}_{j}$ via function $\mathcal{L}$ (line 7) which is described as follows. A *K-*means^35^ clustering model is deployed and is fitted by passing in all ${\overline{y} }_{j}^{\left(m,i\right)}$ of the whole dataset ${\mathbb{D}}$ as its input. The centers of gravities are then returned, with one centroid per class in dataset ${\mathbb{D}}$ being mandated. In other words, each class label is associated with a single cluster represented in the *K*-means resulting activation map. An inter-cluster Euclidean distance *a* from ${{\varvec{x}}}_{j}$ to its gravitational center, and an intra-cluster distance *b* to other class’s center are calculated. The relative noise confident score ${\overline{l} }_{j}^{\left(m,i\right)}=a-b$ is then calculated, where the larger the score is, the more likely that ${{\varvec{x}}}_{j}$ is considered as noise/mislabeled (i.e., located relatively close to the center of other class).

The procedure is repeated for each sample in dataset ${\mathbb{D}}$, and the final label-noise confidence score of ${{\varvec{x}}}_{j}$ is calculated by averaging all results obtained for each model and each cross-validation phase as in line 10. ${\overline{l} }_{j}$ is normalized into the range from 0 → 100 to represent the label-noise confidence score. This method was inspired by the Silhouette Coefficient method, given the relative Euclidean distance from an input sample to different clusters’ centers.

The rest of Algorithm 1 is straightforward when the pre-defined filtering threshold $T$ is utilized to rule out highly suspected noisy samples from the dataset. The threshold $T$ corresponding with the number of noisy samples is practically estimated based on the calculation of number of samples that are misclassified with relatively high predicted confidence scores.

Note that the holdout test set is never involved in the cleansing process, as it has been withdrawn prior to the definition of ${\mathbb{D}}$ and the application of the LDC. Once the LDC is completed and low-quality data excluded to obtain a cleansed dataset, a holdout validation set is also withdrawn. To measure the extent to which cleansing was successful, a final AI model can be trained on the newly cleansed training set, reporting on the cleansed validation set as a reliable measurement of performance. The AI may then be reported on a holdout test set.

### Dataset composition and settings

#### Time-series dataset

The Human Activity Recognition (HAR) dataset^11^ was built from recordings of 30 volunteers, aged 19–48 years, performing several daily activities while carrying a waist-mounted smartphone embedded with accelerometer and gyroscope. The objective is to classify human physical activities into one of six activities performed. In this study, we selected two activities, sitting and standing, to represent a two-class problem. This included 2660 records (1374 with standing ground-truth and 1286 with sitting ground-truth outcomes)^11^. The dataset is expanded to consider multiple classes in a separate experiment, totaling 7352 records (adding 1407 for lying down, 1226 for walking horizontally, 986 for walking downstairs and 1073 for walking upstairs). Each record represented a 561-dimensional feature vector of triaxial acceleration from the accelerometer, and the estimated body acceleration and triaxial angular velocity from the gyroscope. The accelerometer and gyroscope records were pre-processed by applying noise filters and then sampled in fixed width sliding windows of 2.56 s with 50% overlap. From each window, a vector of features was obtained by calculating variables from the time and frequency domain.

This was considered a time-series binary problem since the record’s feature dimension is large. The dataset was naturally very clean with a validation accuracy exceeding 99% for binary problem and 98% for the 6-class problem. To evaluate the LDC approach on this dataset, approximately 30% of synthetic errors were introduced into each class by arbitrarily switching the label of records from one class to the other. For each class this corresponded to approximately 375 flipped labels each.

#### Record-based dataset

The Patient Care dataset^12^ contained a variety of patient laboratory test results such as hematocrit, erythrocytes, and many others, as well as key demographics including the patient’s age and gender. The information in each record was used to determine whether a patient was potentially allocated for inpatient or outpatient treatment at a hospital (forming two target labels of this binary classification problem). The dataset contained 4412 patient records in which 1784 records were labeled as ‘inpatient’ and another 2628 labeled as ‘outpatient’ hospital care. Each record was represented by 10 attributes^12^. This problem is considered inherently difficult compared to the HAR problem, since a total accuracy of only approximately 74% was obtained on the validation dataset when the data was split into training and validation datasets with a ratio of 80% versus 20%, respectively.

The original dataset was divided into two sets, the training and validation set (4012 records: 1643 inpatients, 2369 outpatients) which was then used in the LDC algorithm, and a hold-out test set (400 records: 141 inpatients, 259 outpatients) which was treated as an original uncleaned blind test set. The cleansed training dataset after LDC application was made available for several subsequent model training runs, which were validated on the cleansed validation set (20% of the total cleansed dataset), and also the uncleansed blind test set. These results were collected and compared with the models trained prior to LDC.

#### Image-based dataset

The Oocyte Image dataset^13^ contained 1180 static images of human eggs (oocytes), denuded of cumulus cells and imaged immediately prior to a process known as Intracytoplasmic Sperm Injection (ICSI). Images were provided for 116 consecutive patients treated at a single in vitro fertilization (IVF) clinic—California Fertility Partners, in 2021. Each image contained a single oocyte, with a linked outcome associated with embryo development post-fertilization. This outcome was recorded by the embryologists during the data collection phase of this experiment, and indicated whether the fertilized oocyte had developed into a blastocyst (a specific stage in embryo development) by Day 5, 6 or 7 after IVF. There were 483 (40.9%) oocytes that developed into a blastocyst (labeled as ‘competent’), and 697 oocytes that did not (labeled as ‘incompetent’).

Prior to any removal of records associated with errors, a validation set of 236 images was specified, with 95 oocytes (40.2%) that developed into a blastocyst after IVF (competent), and 142 that did not develop into a blastocyst after IVF (incompetent), representing an almost identical class ratio to the whole dataset.

In addition to the outcome label, each record also included associated metadata. Each data entry also specified whether male infertility factors were identified during treatment. 441 images were associated with treatments involving male infertility factors (37.4%), and 739 images had no such infertility factors associated with them (62.6%).

UDC and LDC methods were then applied to the 739 images without male infertility factors using a 19.9% cleansing threshold. Several subsequent models were trained and validated on the resulting cleansed dataset, consisting of 592 images. Experimental results were compared using evaluation metrics: total accuracy, sensitivity, and specificity, between UDC and LDC methods and the models prior to any data cleansing techniques. The final dataset included 500 training images and a validation set of 92 images.

### Ethics approval

The Oocyte Image dataset was collected prospectively in a non-interventional study. The study was approved by the Ovation Research Ethics Committee (Newport Beach, CA 92,663, USA), with accession number OF210913B.

## Results

In this section, a one-dimensional convolutional neural network model (1D-CNN)-based architecture was developed for use with record-based datasets. It employed a label-clustering deep learning method for data cleansing ("Label-noise confidence algorithm" section), and was compared to results without cleansing. Convolutional neural network models were originally developed for image classification problems^36^, however, these can be customized to one-dimensional sequences of data, as shown for the datasets below. The models learn to extract features from sequences of observations, and how to map the internal features to different class labels. The benefit of using 1D-CNN layers for record-based classification is that they can learn from the raw feature data directly, and hence do not require domain expertise to manually engineer any input features.

In this study, the label-clustering-based approach LDC was validated on two contrasting record-based binary classification problems. Firstly, a HAR accelerometer time-series dataset was considered, with two classes selected for analysis—sitting and standing. This dataset was considered good quality, with a high proportion of correct labels, resulting in validation accuracies exceeding 99%. Secondly, a Patient Care record-based dataset was also considered, for which the two classes were ‘inpatient’ and ‘outpatient’. This dataset contained a higher degree of label noise, and was considered a hard ML problem in the absence of a viable data cleansing method. Both of these datasets are described in "Dataset composition and settings" section.

Finally, label-clustering was applied to an image-based classification problem—a set of denuded human oocyte images taken prior to ICSI. This was also a binary classification problem, for which the two classes were defined by whether the fertilized oocyte developed into an embryo (blastocyst) or not—these were labeled as ‘competent’ or ‘incompetent’ respectively. The technical aspects underpinning these experiments will be presented accordingly.

### Comparing label-clustering with different approaches for human activity recognition

#### Comparing with a consensus approach to binary classification

LDC was used to cleanse a version of the HAR dataset where synthetic errors were intentionally injected by flipping approximately 30% of the labels for each class. This injection of synthetic errors allowed the ground truth label to be tracked, so that detection of errors by LDC could be quantitated in order to calculate the IoU score or the error identification accuracy. Noise or errors in the dataset typically deteriorate the model training process and reduce AI accuracy. Therefore, the increase in accuracy after errors were removed by LDC was also used as a measure of data cleansing performance.

**Figure 2:**
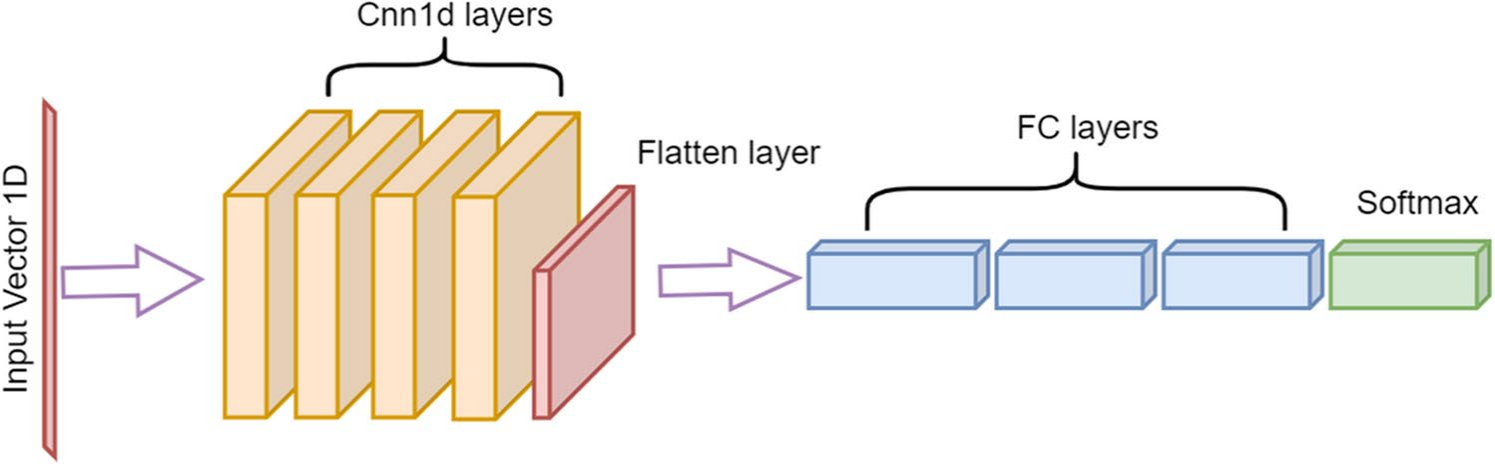
Illustration of a record-based classification model. FC denotes full-connected neural network layers. Softmax denotes a layer that applies the softmax function to the output of the final FC layer. Technical details of this architecture are described in Section "Experiment design". This architecture was used for both HAR and Patient Care record-based classification problems.

To begin the process, 15 AI models were trained consisting of 3 network architectures, namely the single input branch MLP with two fully connected layers of size 150 and 30, three input branch MLP (each input branch is characterized with an activation function from Sigmoid, Tanh and ReLU functions) with two fully connected layers of size 300 and 128, and an 1D-CNN with 1 channel, kernel size 400 and fully connected layer size 30. fivefold cross validation was performed for each architecture.

as described in "Experiment design" section, with the best epoch chosen on each model’s validation set according to log loss as the preferred evaluation metric. An illustration of a 1D-CNN for record classification is shown in Fig. 2. It should be noted that by setting the number of CNN layers to zero, 1D-CNN practically turns into an MLP.

**Figure 3:**
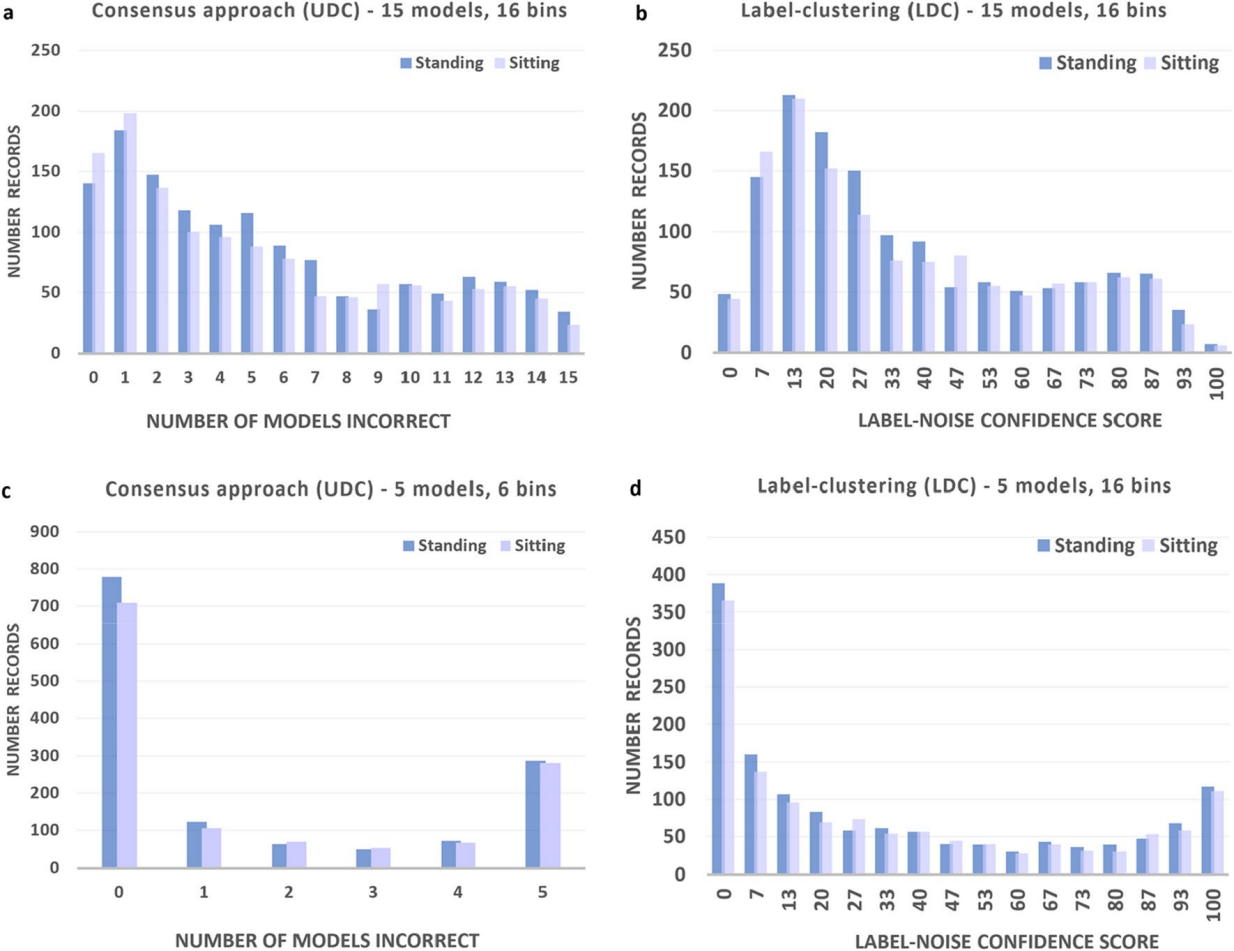
Histogram comparisons for the HAR dataset with 30% injected errors at various bin sizes, for both sitting and standing classes associated with a binary classification problem. (**a**) UDC using all 15 models, (**b**) LDC using all 15 models, (**c**) UDC using the 5 top-performing models based on log loss, and (**d**) LDC using the 5 top-performing models based on log loss.

Figure 3 compares histogram outputs of records analyzed by both LDC and UDC, the original, consensus-based approach to data cleansing^1^. The UDC histogram was produced by aggregating the number of models that incorrectly predicted the label for each datapoint. Figure 3a shows UDC histogram results for all 15 models, and Fig. 3c shows results for the 5 top-performing models. The number of bins within these two histograms is constrained by the number of models voting on each datapoint. The UDC histograms in Fig. 3a,c can be interpreted as follows. Datapoints that were misclassified by a comparatively high number of contrasting models are considered more likely to be mislabeled. The number of models (*n*_*i*_) reaching a consensus that the datapoint $i$ is mislabeled taken as a score represented by the $x$-axis, in these two Figures. If a datapoint corresponds to a moderate score (e.g. 7–8 out of 15 in this case) it is considered likely noisy data, meaning that it is unclear if it is mislabeled or not. The datapoints with a low consensus that they are mislabeled are retained for training. Note that the cleansing ‘threshold’, that is, the number of models needed to be considered mislabeled or noisy, can be adjusted to change the bin-width of the histogram. A lower threshold will capture more mislabeled or noisy data, however, it will also mistakenly identify more correct data as being mislabeled or noisy. The tradeoff between these are captured inherently in the definition of the IoU and the error identification accuracy—see "Experiment design" section. Setting cleansing thresholds is discussed futher below.

Figure 3b,d show equivalent LDC histograms produced by introducing label-clustering. Note that the x-axis is now a probabilistic, continuous score that can be binned at the desired level of precision, rather than being constrained by the number of models voting on each datapoint. This label-noise confidence score ranges from 0 (most confident in the label) to 100 (least confident in the label). It is interpreted similarly to the UDC histogram, with very high and moderately high label-noise scores indicating the data is likely mislabeled or noisy, respectively.

When using all 15 models, the shape of the UDC and LDC histograms were superficially similar. However, it is important to note that the bin size for the label-noise confidence score can arbitrarily be made finer using the LDC method, allowing for more precise thresholds of noise tolerance to be selected. This is especially apparent when considering the top 5 models in Fig. 3c versus d, where a more finely grained shape was resolved in the case of the label-clustering result (Fig. 3d), allowing for more precise identification of errors.

Use of only the 5 top-performing models led to a cleaner histogram shape in both cases, indicating an improved accuracy for identifying errors. This is consistent with excluding the more poorly performing models, which would have otherwise compromised the ability for error detection. Note that, in order to produce a finer histogram for the UDC consensus approach, more models would be required and the training cost would therefore increase accordingly. The LDC method on the other hand is able to generate a finer histogram with fewer models, reducing the training burden while maintaining the improved precision for selecting cleansing thresholds.

**Figure 4:**
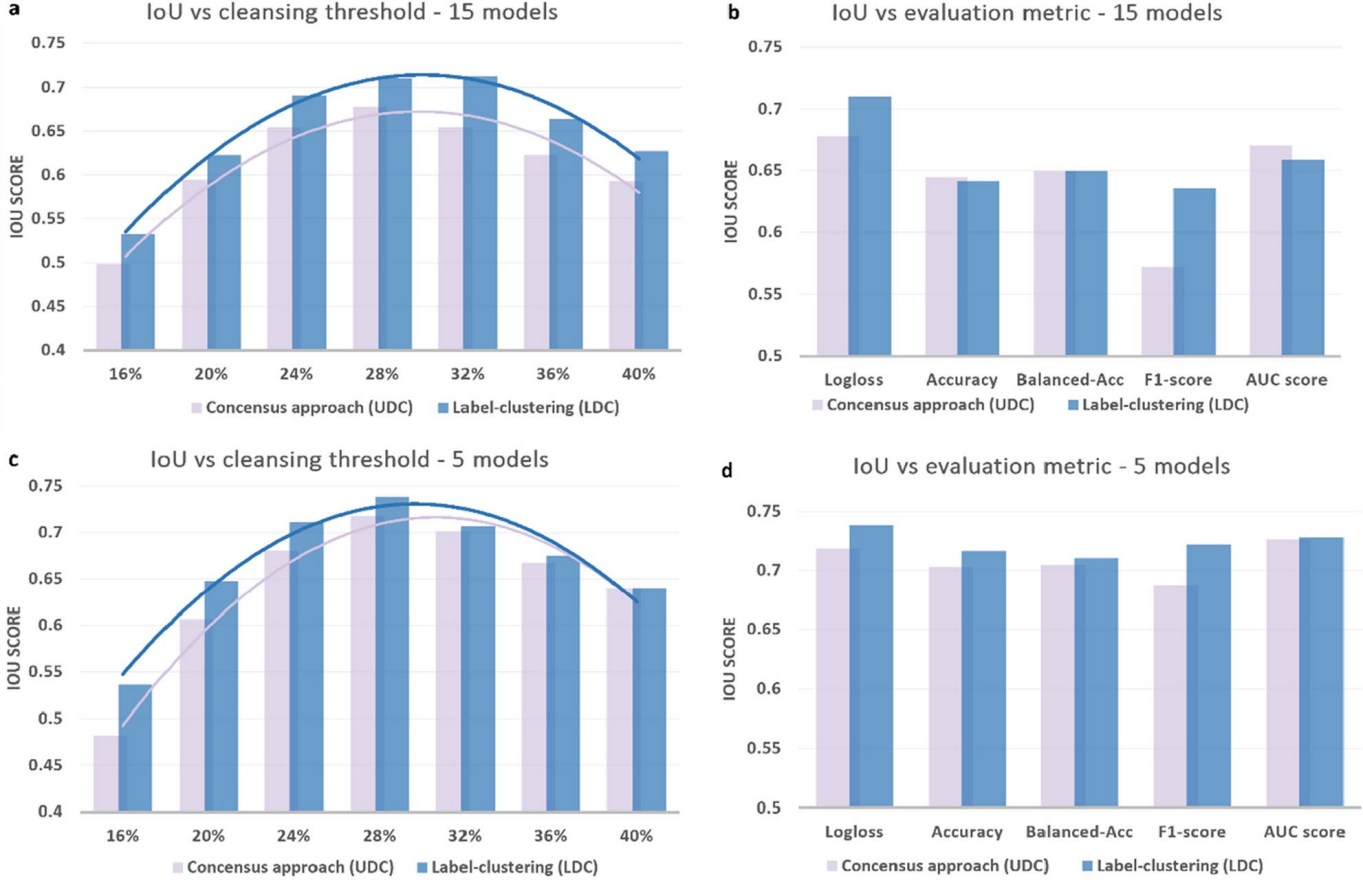
IoU scores associated with UDC and LDC for the HAR dataset (30% injected errors). (**a**) IoU scores were compared for different cleansing thresholds associated with the percentage of datapoints removed using log loss as the metric for evaluating the best epochs for all 15 models. (**b**) IoU scores were compared for different metrics for evaluating the best epochs for all 15 models, with a fixed cleansing threshold of 28.2%. (**c**) IoU scores were compared for different cleansing thresholds using log loss as the evaluation metric for the 5 top-performing models. (**d**) IoU scores were compared for different evaluation metrics for the 5 top-performing models, with a fixed cleansing threshold of 28.2%.

Figure 4a and c show how the IoU score changed with respect to the cleansing threshold chosen based on the percentage of datapoints removed for both UDC and LDC. Log loss was used as the metric for evaluating the best epochs for all 15 models (Fig. 3a) or for the 5 top-performing models (Fig. 4c). With a 30% noise level in the dataset, removing a more conservative percentage of records (e.g. 16%) reduced the IoU for both UDC and LDC. Similarly, aggressively removing records at a rate of 40% also reduced the IoU for both UDC and LDC. In practice, the amount of noise is not known in advance, and so the cleansing threshold can be obtained heuristically using the corresponding histograms.

Figure 4b,d show how the IoU score changed with respect to the metric used to evaluate the best epoch for each model. A fixed cleansing threshold of 28.2% was used (similar to the amount of injected errors at 30%) for all 15 models in Fig. 3b, and only the 5 top-performing models in Fig. 4d. Results showed that use of the 5 top-performing models was less sensitive to the choice of metric (due to the clean shape of the histograms presented in Fig. 3c,d), however, log loss outperformed the other evaluation metrics when worse-performing models were included in generating the histograms.

The IoU score reached a maximum value of 74% for both LDC and UDC. However, it is worth noting that the IoU score underestimates the performance of the cleansing procedures since it penalizes the removal of correct records and the retaining of errors to the same extent (see "Experiment design" section). For large datasets, the removal of correct records generally does not hamper AI performance in the same way as the presence of errors^1^. Therefore, an Error Identification Accuracy (EIA) measure should instead be used to evaluate the percentage of records that were correctly identified as errors and removed (defined in "Experiment design" section).

**Figure 5:**
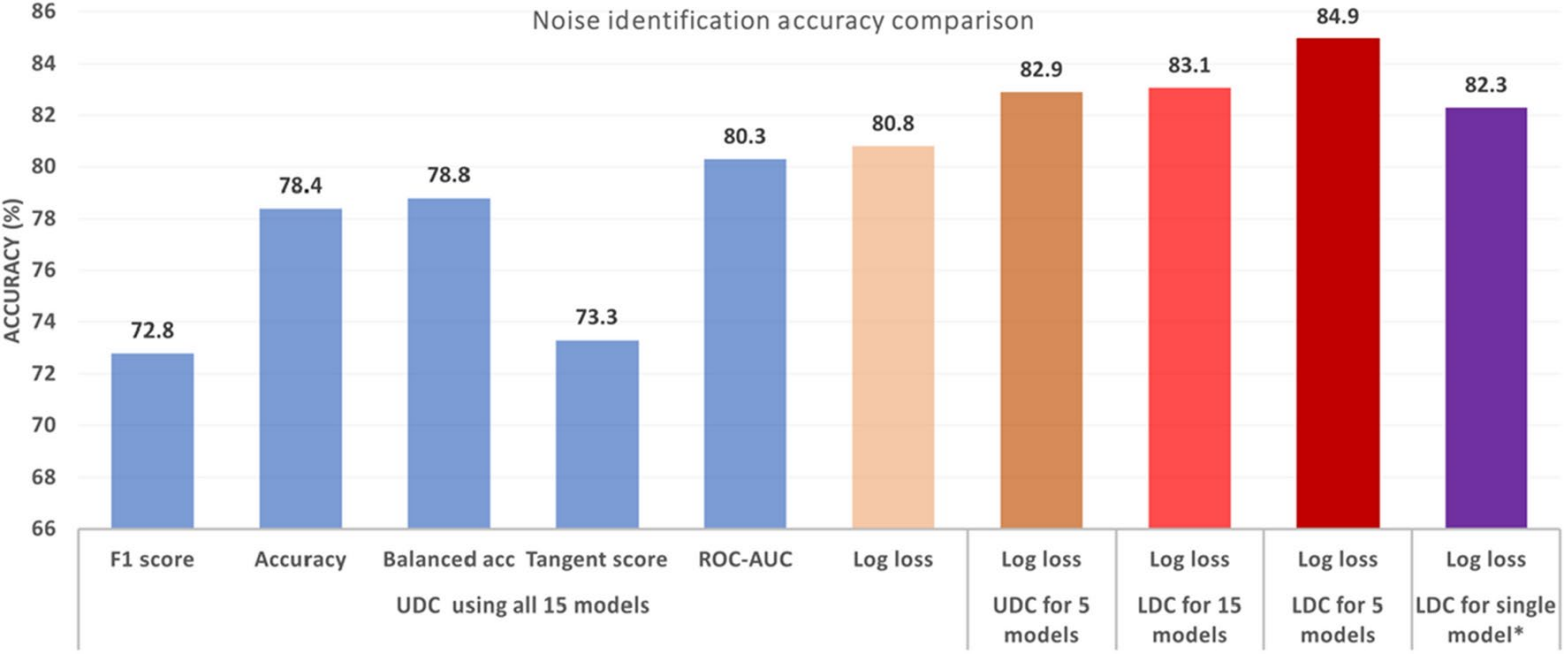
Comparison of error identification accuracies for UDC and LDC on the HAR dataset (30% injected errors), using all 15 models or the 5 top-performing models, and using a range of metrics for evaluating the best epoch for each model (F1 score, total accuracy, balanced accuracy, tangent score^37^, Area Under the Receiver Operating Curve (ROC-AUC) and Log loss). *LDC applied to a single model must exclude the validation set used to select the best epoch.

For a fixed cleansing threshold of 28.2%, the accuracies of UDC and LDC for identifying errors are shown in Fig. 5. The maximum performance of UDC on all 15 models was 80.8% using the log loss metric. The EIA increased to 82.9% using only the 5 top-performing models. LDC, however, is completely deterministic with a continuous scale, leading to a more reliable and precise method for identifying errors more efficiently (less models needed and reduced computing cost). The performance of LDC using only the 5 top-performing models was therefore even higher at 84.9%.

The choice of how many hyperparameter options should be selected, and thus how many models should be used to contribute to an application of UDC or LDC is a tradeoff between computational resources and robustness of determination of confidence that data are mislabeled or noisy. For any given new problem, the models generally should be selected from the highest performing models available.

Interestingly, LDC can even be applied to a *single* model, representing the model’s own measurement of the score self-consistency, noting that the validation set used to select the best epoch must then be excluded (since *k*-fold cross validation cannot be used for a single model). Label-clustering from the self-consistency of a single model on a dataset could still achieve an error identification accuracy of 82.3% but with a compute resource reduction of 93% compared to the 15-model UDC approach^1^.

So far, the LDC method has been applied to a binary classification problem. Typically, HAR analysis requires more than two categories to be of real-world use. The LDC method may be extended to consider multi-class classification, in the same manner as previously explored for the UDC consensus approach^1^.

#### Comparing with a consensus approach to multi-class classification

**Figure 6:**
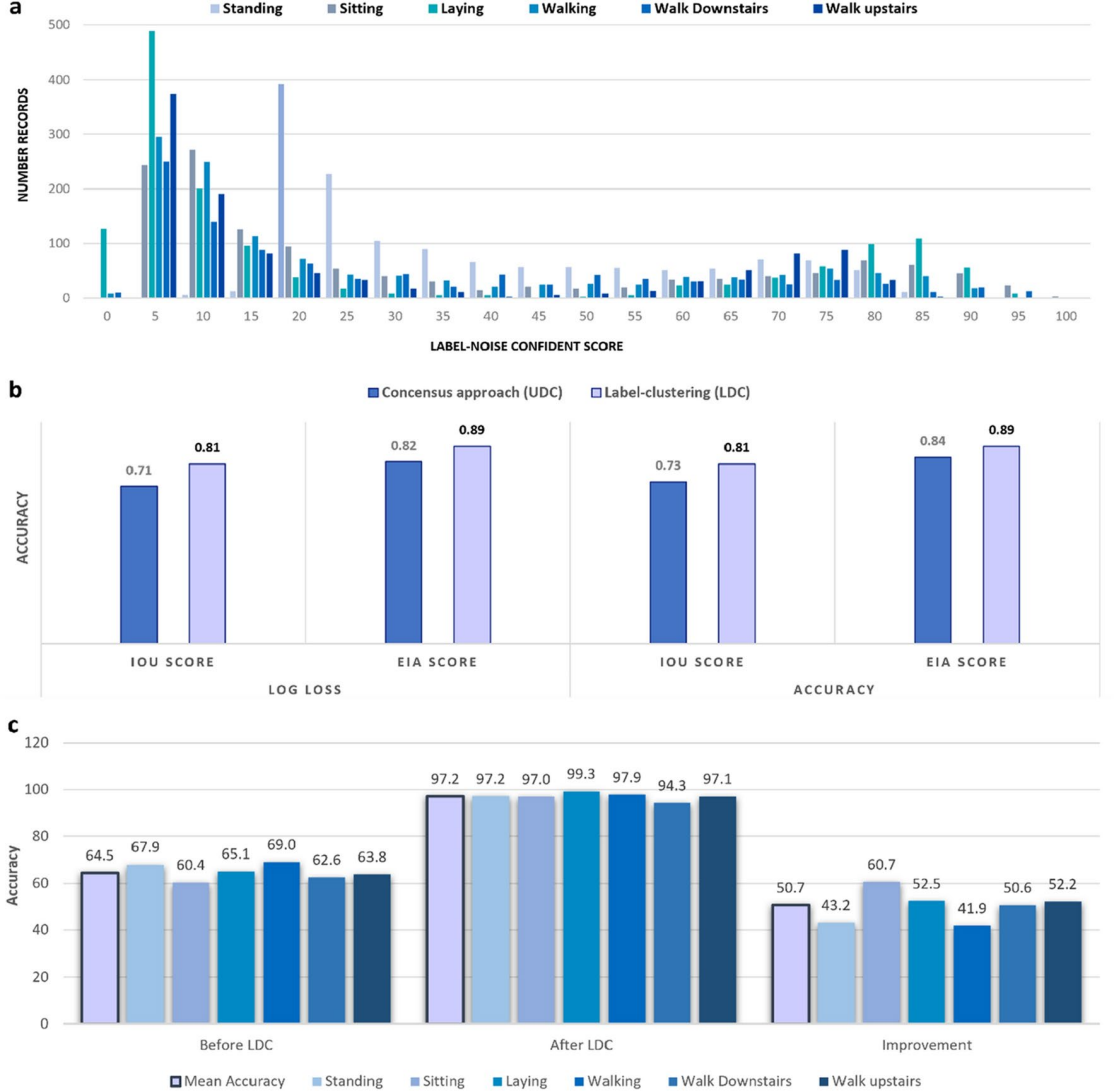
Model comparison for the HAR multi-class dataset (30% injected errors). (**a**) Multi-class problem LDC histogram. (**b**) IoU and EIA scores were compared for different cleansing methods using log loss and accuracy as the metrics for evaluating the best epochs for 5 models. **c.** The validation results for the multi-class HAR dataset with 30% errors injected are compared between training models on the noisy dataset (before LDC) and when training the models on LDC cleaned dataset.

The LDC method can be applied to multiple classes, as demonstrated on an extended HAR dataset, with 6 classes as described in "Time-series dataset" section. Similar to the binary case, 30% noise is injected into the dataset by changing the label of 30% of the dataset to a randomly-selected other class. Figure 6a presents the histogram associated with label-noise confidence scores for each datapoint, divided into ground-truth labels. While some classes contain a high proportion of clearly labeled and clearly mislabeled data (e.g. lying down), others contain a greater proportion of noisy or ambiguous data (sitting). Regardless of using log loss or total accuracy as the metric for selecting the best epoch of each constituent model, LDC always achieves higher IoU and EIA scores by at least 6% (from 0.84 to 0.89 in EIA score when using total accuracy) and by up to 14% (from 0.71 to 0.81 in IoU score when using log loss) as shown in Fig. 6b. Figure 6c shows an increase in total accuracy from 64.5 to 97.2%, representing a 50.7% improvement for models trained on the LDC-cleansed dataset with a maximum improvement for sitting class representing a 60.7% improvement.

#### Comparing with a robust loss function approach to binary or multi-class classification

In this section, a comparison is made between LDC and other state-of-the-art approaches, namely a generalized cross entropy noise-robust loss function (GEN) that can be seen as a generalization of mean absolute value and categorial cross entropy loss^38^, and another symmetric cross entropy function (SYM) that boosts cross entropy loss symmetrically with a counterpart ‘reverse cross entropy’^39^. To maintain a fair comparison, GEN and SYM methods were reported on a cleansed test set which accounts for 20% of the whole original HAR dataset prior to noise injection. The training set is injected with 30% noise in the same way as in "Comparing with a consensus approach to binary classification" section. All model parameters and experimental settings were kept consistent with the models trained post-LDC. While LDC takes an intermediate step to remove mislabeled or highly noisy samples before performing a post-LDC training on the cleansed training set, these other methods are purported to be more robust to noise without requiring a data cleansing step. Table 1 presents total accuracies on HAR binary and multi-class problem for different approaches. The best accuracy for any epoch reported on the test set, and last epoch accuracy are both shown in order to compare the models’ performance and their robustness against overfitting. For the binary HAR problem, LDC reported a minimum of 10.2% improvement compared to these novel loss functions. SYM is seemingly more volatile than GEN since the model based on SYM attained a higher “Best” accuracy, but deteriorated sharply during training, resulting in much lower “Last” accuracy. For the multi-class HAR problem, a similar trend can be observed, but the difference in accuracy using data cleansing is less, with a 0.2–6.3% improvement.

**Table 1:** Comparing performance metrics of different approaches for binary HAR and multi-class HAR classification problem using two scenarios: “Best” accuracy overall and the corresponding “Last” epoch’s accuracy of the same model.

Method	Binary classification		Multi-class classification	
Epoch selection	Best accuracy	Last epoch	Best accuracy	Last epoch
GEN^37^ metrics	89.8, 89.5, 90.2, 89.8	72.2, 72.6,72.5, 72.5	97.6, 97.7, 97.8, 97.7	97.3, 97.4, 97.3, 97.4
SYM^39^ metrics	90.6, 90.5, 90.6, 90.5	68.0, 68.3, 68.2, 68.2	98.0, 98.1, 98.2, 98.1	92.0, 91.9, 92.0, 92.0
Post-LDC metrics	99.8, 99.8, 99.8, 99.8	99.8, 99.8, 99.8, 99.8	98.2, 98.2, 98.2, 98.2	97.8, 97.8, 97.7, 97.7

The metrics are reported in the following order: total accuracy, precision, recall and f1-score, expressed as percentages.

### Application of LDC to HAR and patient care

In this section, the LDC method was applied to remove errors from the HAR datasets comprising 30% injected errors, and from a Patient Care dataset that contained inherent errors. The resulting AI models trained on these LDC cleansed datasets were then evaluated for performance improvement compared to models trained on the uncleansed datasets.

Histograms for the HAR dataset following LDC are shown in Fig. 3. The cleansing threshold of 28.2% was applied for AI training.

**Figure 7:**
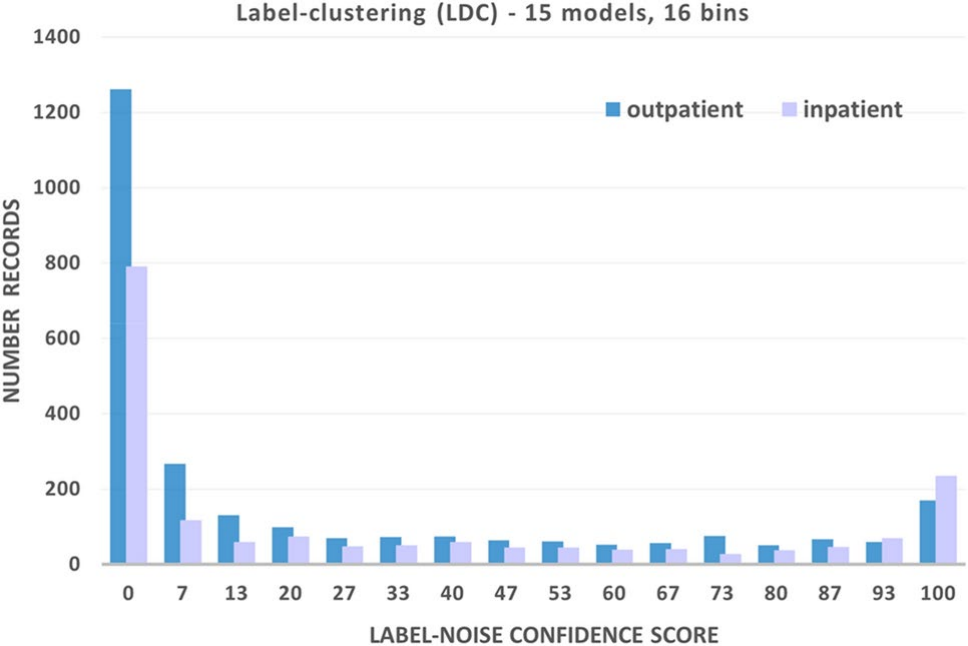
Label-noise confidence score histograms for the Patient Care dataset for both inpatient and outpatient classes for a binary classification problem, using 16 bins in each case.

The Patient Care dataset is considered a hard ML problem with a high proportion of label errors. In this case, synthetic label changes are not used, and the dataset contains realistic errors that have arisen naturally in data collection. 15 models were used in applying LDC to this dataset, and the resulting histograms for both inpatients and outpatients are shown in Fig. 7. In general, the choice of cleansing threshold is a tradeoff that is highly problem-dependent. How aggressively one is willing to cleanse the dataset will depend on the ease of the problem from an ML perspective, and the amount of data available to cleanse, so that it does not fall below a reasonable amount required to train a model. As a general rule, the threshold can be obtained heuristically by assessing a range of different thresholds and maximizing the value of the IoU score or EIA, as described in "Comparing with a consensus approach to binary classification" and "Comparing with a consensus approach to multi-class classification" sections.

After LDC, 1323 samples (623 inpatients and 700 outpatients), equivalent to 30.0% of the dataset, were identified as erroneous (mislabeled) and removed. 186 of these mislabeled records received a label-noise confidence score greater than 99.99%. This left 3,089 records for training (1161 inpatients, 1928 outpatients), and 400 (141 inpatients, 259 outpatients) uncleansed images were held back as a noisy test set.

**Figure 8:**
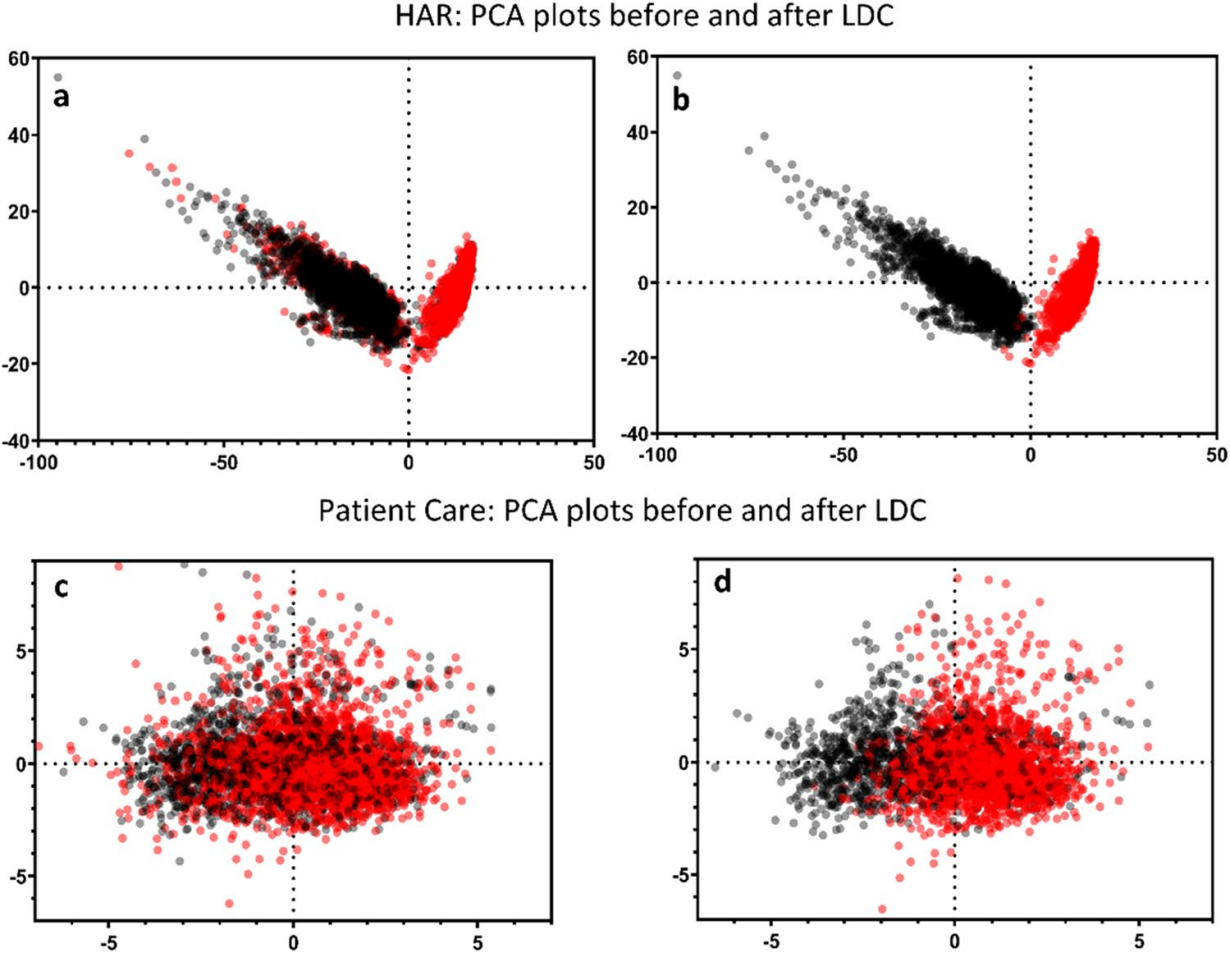
2-d maps generated from the first two components of a PCA applied to the model scores used as input. (**a**) The binary HAR dataset with 30% injected errors was plotted prior to the removal of samples identified using LDC at a 28.2% threshold. Black samples correspond to ‘sitting’ and red samples correspond to ‘standing’. (**b**) The same HAR dataset was plotted after the removal of the samples identified using LDC. (**c**) The Patient Care dataset was plotted prior to LDC sample removal at a 30.0% threshold. Black samples correspond to ‘inpatient’ and red samples correspond to ‘outpatient’. (**d**) The same Patient Care dataset was plotted after the removal of the samples identified using LDC.

To confirm that the data identified for removal using the LDC approach represent predominantly mislabeled data, rather than merely hard-to-classify edge cases, the histogram of Fig. 7 shows a peak associated with a high level of label-noise confidence score, indicating that there is a high level of agreement among the LDC contributing models that these data are consistent with the opposite class. To further shed light on the apparent mislabeling, the distribution of both the binary HAR and Patient Care datasets before and after removal of samples identified by LDC can be visualized using Principal Component Analysis (PCA)^40^. Figure 8 shows the 2-d maps generated from the first two PCA components applied to the model scores. Figure 8a demonstrates how errors contaminated clusters of samples corresponding to the two classes of the HAR dataset, sitting and standing. Figure 8b shows how cleanly the classes are separated after LDC. The equivalent comparison for the Patient Care dataset is shown in Fig. 8c,d. The separations between the two classes, inpatient and outpatient, are less distinct, clearly indicating the difficulty of the classification problem for the ML models described in "Experiment design" section.

**Figure 9:**
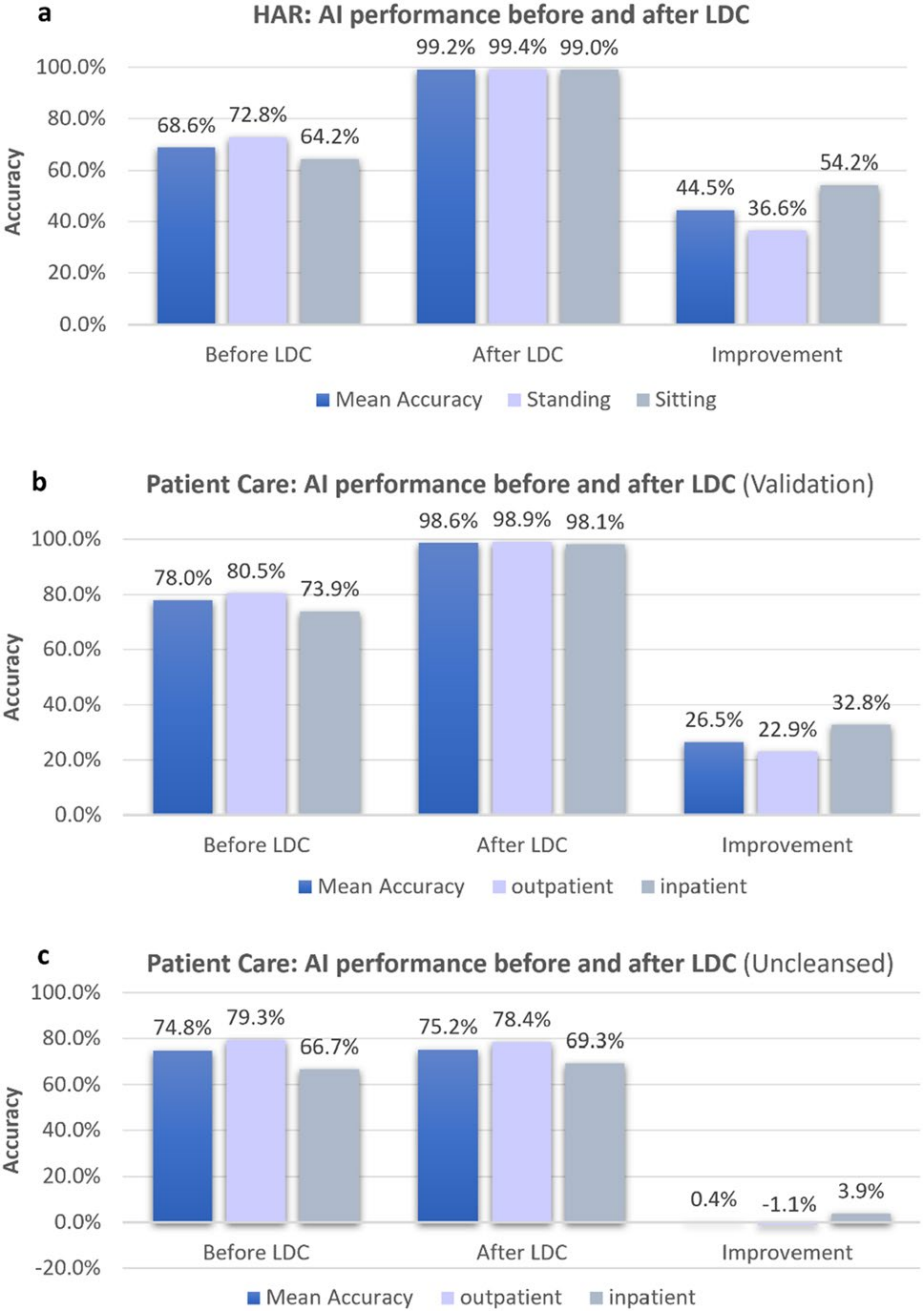
Average performance of three ML models trained prior to LDC applied, measured using total accuracy and class-wise accuracies, and their respective improvements over models trained without LDC applied. (**a**) The results for the HAR dataset with 30% errors injected are shown. (**b**) The corresponding results for the Patient Care Validation dataset are shown. (**c**) The results for the Patient Care blind test set are shown, where the models both pre- and post-LDC were applied to an uncleansed (real world) version of the test set.

Figure 9 shows the performance of ML models trained on these two datasets measured using total accuracy and class accuracies, and their corresponding improvements demonstrated after LDC. For each dataset, three ML models were trained and the performance for each class was averaged. In the case of the HAR dataset, Fig. 9a shows an accuracy improvement from 68.6 to 99.2%, representing a sizeable improvement of 44.5%. While the sitting class accuracy was lower than the standing class accuracy prior to LDC, the improvement was more significant, indicating that the LDC had successfully identified more errors in the sitting class. This results in very similar final accuracies of 99.4% (standing) and 99.0% (sitting). This demonstrates that the LDC is indeed removing erroneous data, and hence improving the quality of the training data.

Figure 9b shows an increase in total accuracy from 78.0 to 98.6% on the Patient Care Validation dataset, representing a 26.5% improvement for models trained on the LDC-cleansed dataset. Similar to the HAR dataset, one class performed more poorly (inpatient) but also exhibited a greater improvement (32.8%). The final class accuracies after LDC had been applied were therefore also similar at 98.9% (outpatient) and 98.1% (inpatient).

While the Validation dataset indicated a sizeable uplift in performance for AI models trained using LDC, it is, by definition, a cleansed dataset, so it is also important to quote the accuracies of the models applied to an uncleansed blind test set. This is more representative of a real-world clinical setting, with inherent errors contained in the data. Interestingly, the AI accuracy results on an uncleansed test set were similar with and without LDC applied (Fig. 9c), even though the results on the cleansed Validation dataset (Fig. 9b) showed that AI performance had improved when using LDC. This observation highlights how challenging it is to define the true performance of an AI model when measured on a blind test set taken from a real-world clinical setting, where inherent errors exist. AI accuracy can only be truly measured using an error-free dataset, which is the reason we took the predominantly clean HAR dataset, and injected known errors, to test the performance of LDC for removing these errors.

### Application of LDC to labeled oocyte images

In this section, a denuded human oocyte image dataset was taken as input to the UDC/LDC algorithms, building on recent results of a prototype Oocyte Assessment model^13^. The images were labeled based on whether the oocyte, upon IVF using ICSI, developed to the blastocyst stage, which is a key marker of oocyte competence. Inherent label errors associated with this dataset emerge from confounding biological factors, such as male infertility that can affect blastocyst development (e.g. abnormal sperm morphology or motility). Details of the oocyte image dataset are presented in "Dataset composition and settings" section.

**Figure 10:**
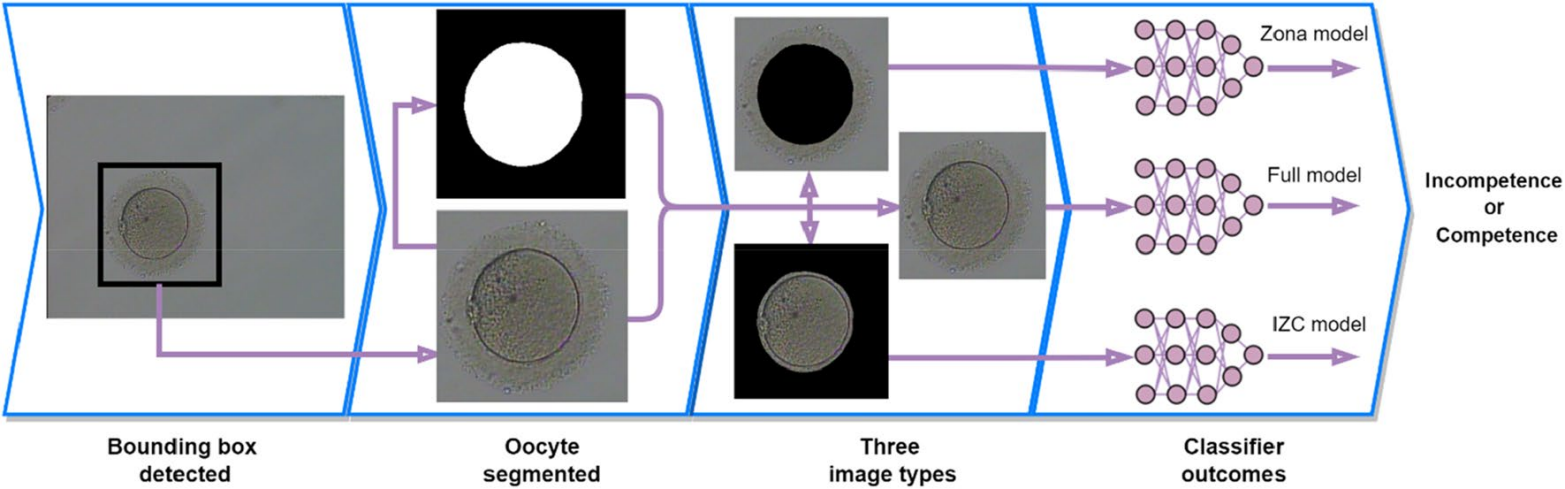
Workflow for pre-processing and training ML models on an image-based oocyte classification problem. Each image was run through a trained Faster R-CNN model to obtain a bounding box. After cropping to the bounding box, the image was optionally segmented using a U-Net model with a ResNet-50 encoder, masking either the intrazonal cavity, or the *zona pellucida* and background regions. Binary classification models were then trained on a given labeled dataset, based on a range of architectures and hyperparameter values.

The network architectures used for the LDC process for this image classification problem are described in "Experiment design" section. Figure 10 presents the workflow or the process of predicting or identifying an oocyte image as incompetent or competent. To develop a robust prediction algorithm, image preprocessing steps are essential prior to training a deep learning classification model. First, oocyte images underwent pre-processing using a trained, Region Based deep convolutional neural network (Faster R-CNN)^27^ to obtain bounding boxes for each oocyte. The images were then cropped to the bounding box before optionally feeding the image into a segmentation model especially trained on oocyte images, using a U-Net architecture^29^ with a ResNet-50 encoder^28^. In a similar manner to embryo viability classification^41,42^ and PGT-A classification problem^43^, segmented images contained masks of either the intrazonal cavity (IZC), or the *zona pellucida* region and background (see Fig. 10, third panel). Models trained on these inputs were denoted IZC Models and Zona Models respectively.

After pre-processing, a range of deep learning architectures and hyperparameter values were used to train binary classification models on a labeled set of oocyte images. The dataset was curated using the LDC process, and the results before and after LDC compared.

Using the oocyte dataset defined in "Dataset composition and settings" section with the outcome label defined by whether the oocyte developed into a blastocyst, a naïve application of a range of deep learning models for binary classification training (without UDC or LDC) led to a total accuracy of 61.8%. This was reported as an average performance using 5-fold cross validation, with the validation sets containing 236 images, using architectures summarized in "Experiment design" section.

**Figure 11:**
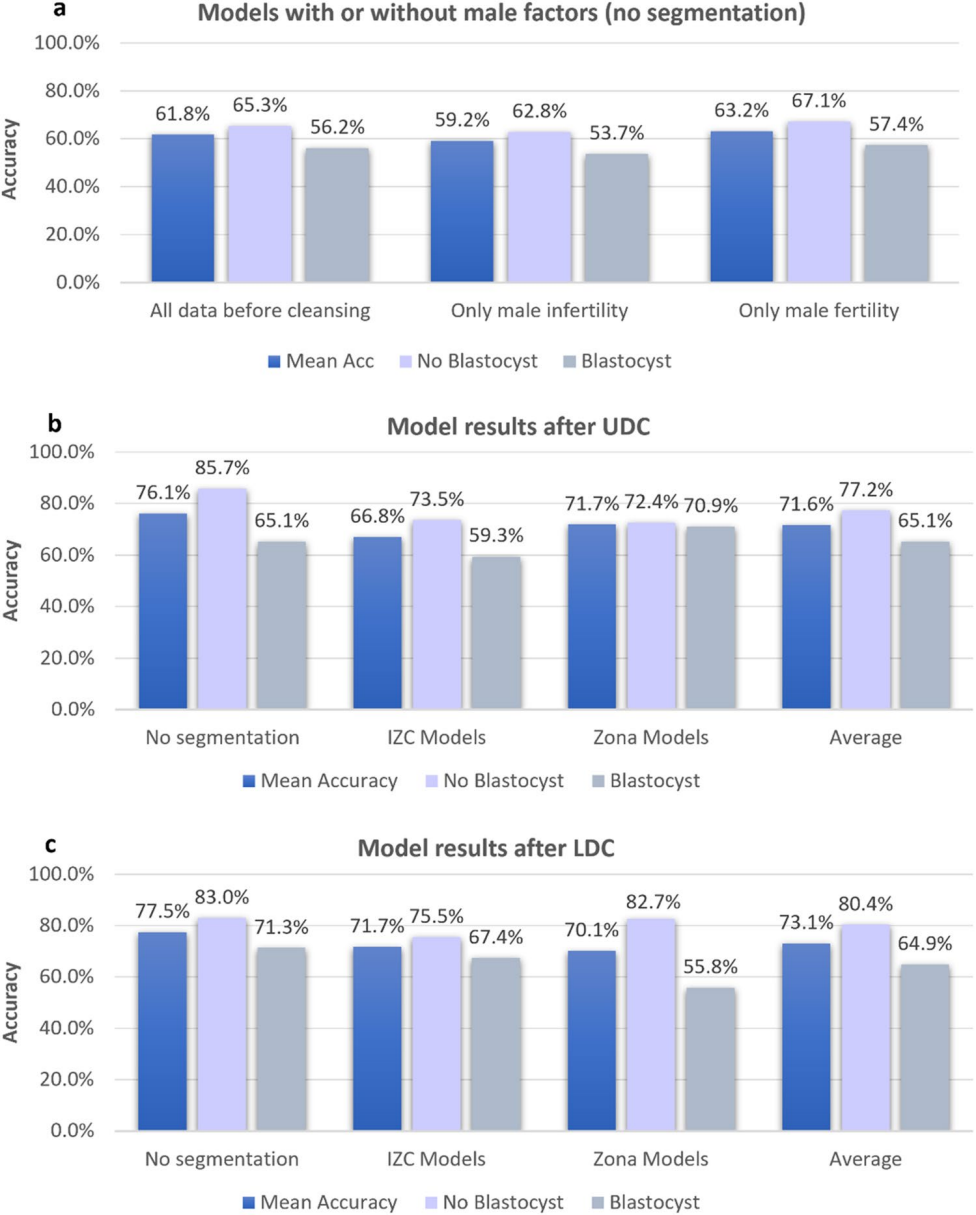
Comparison of ML model performance in terms of total accuracy and class-wise accuracies for identifying oocytes that did not lead to a blastocyst (incompetent) and oocytes that did lead to a blastocyst (competent). (**a**) The results prior to removal of confounding factors, and prior to UDC/LDC, are shown on a test set of 236 images with a similar class ratio to the full dataset. The results are quoted on subsets of the dataset which only contain images with male infertility factors, and which contain images with no male infertility factors, respectively. (**b**) After cleansing using UDC, the mean performance of subsequently trained ML models is shown for three kinds of segmentation pre-processing and the average results. (**c**) Corresponding results after data cleansing using LDC.

Removal of images that were linked to male infertility factors from the validation dataset resulted in an improvement beyond this baseline accuracy to 63.2% (Fig. 11a). Correspondingly, performance of the models on data containing only images with associated male infertility factors dropped to 59.2%. Male infertility factors were thus confirmed as a confounding biological variable in this dataset, as they can hinder the development of a blastocyst in cases of otherwise ideal oocyte quality.

The proportion of errors in the dataset appeared relatively high, as evidenced by the ML models incorrectly classifying 36.8–38.2% of the validation images on average. To train ML models appropriately, such that they do not erroneously learn that a high-quality oocyte will not lead to a blastocyst, it was necessary to remove these mislabeled records from the training set. Application of UDC and LDC independently identified 31.6% of the known male infertility cases as mislabeled. These results suggest that the cleansing methods are correctly identifying male infertility factors as a major source of errors for evaluating oocyte competency. Hence, all records associated with male infertility factors were removed prior to applying the UDC and LDC algorithms for AI training, which accounted for 37.4% of the dataset.

The consensus-based UDC approach was then applied to the remaining images to remove another 19.9% of likely mislabeled or noisy data, such that the remaining, cleansed dataset consisted of images anticipated to have high quality labels suitable for training. Note that in this case, a blind test set was not available due to an insufficient initial dataset size. A similar procedure was followed using the label-clustering LDC.

After applying UDC and LDC, subsequently trained ML models were divided into three categories: those without segmentation pre-processing, Zona Models, and IZC Models (see Fig. 10). The performance of these models is shown in Fig. 11b,c for LDC and UDC, respectively. The results were the average over at least two model-training runs for three different deep learning architectures.

Across all segmentation types, an average uplift of 18.4% in total accuracy was observed for the LDC models (61.8% and 73.1% for pre- and post-LDC models, respectively). Furthermore, LDC models showed an average improvement of 2.2% over UDC models. The poorest performing segmented models were the IZC models, which occlude the *zona pellucida* region. Conversely, the unsegmented images performed best, with an average uplift of 25.5% to a total accuracy of 77.5%.

## Discussion

Increasingly efficient algorithms for the accurate automated identification of errors in data can aid in the development of more robust and scalable AI models. This is particularly significant for medical datasets, which are inherently poor quality, where subjectivity, complex biological confounding variables, and data privacy considerations can make manual identification unfeasible.

A novel approach combining deep learning and label-clustering, LDC, demonstrated vastly improved computational efficiency (up to 93% fewer resources required) compared to the UDC method, which is a model-consensus-driven approach^1^. At the same time, the LDC approach retained the ability to identify errors in datasets a priori, that is, through self-consistency alone rather than pre-training on specific instances of known errors.

The method demonstrated improved scalability and a reduced complexity of data management compared to that required to build a consensus-based histogram, replacing it with a continuous label-noise confidence score. Its ability to identify errors was not only maintained, but improved to an accuracy of up to 85%. ML models trained using a cleansed dataset based on Human Activity Recognition, originally containing 30% manually-injected errors, exhibited greater stability during training than models trained on the uncleansed dataset, in that the loss function during training remains flatter, with minimized oscillations that occur from training with noise injected. These models also demonstrated an uplift in total accuracy of up to 45%, from 69 to over 99%. The introduction of a continuous label-noise confidence score, based on label-clustering, allows for precision in removing errors from the dataset for a given threshold or tolerance, and subsequent development of higher performing models.

When extending to a multi-class problem, an even greater accuracy uplift was measured when using this data cleansing method—up to 64.5% improvement. Interestingly, when these results were compared to existing methods for handling noise, namely two choices of robust loss functions for handling data errors, the LDC method showed at least 10% improvement in binary classification, but only comparable or slightly increased improvement in multi-class classification. This might indicate that a multi-class problem is more amenable to methods of handling errors, since there is a greater choice of classes for errors to be distributed into. The accumulation of errors in a binary classification problem is hence likely to be more severe, as the errors conspire to appear systematic, i.e. it ‘looks’ like a rule that is difficult to prevent the AI from learning.

When LDC was applied to a dataset of Patient Care records with a high proportion of inherent errors, it demonstrated an ability to more cleanly separate the classes (inpatient and outpatient), as illustrated by 2-d maps based on the PCA analysis. After removing a high proportion of suspected errors the trained AI model performance again approached 99%, compared to 78% prior to cleansing.

We also highlighted the challenges of testing AI performance on a poor-quality dataset that can be encountered in a real-world clinical setting, and how it can be misleading as to the true performance of AI models. Better performing AI models can show little to no accuracy improvement when applied to datasets with errors.

Turning attention to the medical imaging dataset of denuded human oocytes labeled with outcomes based on blastocyst formation, cleansing was able to identify 31.6% of mislabeled images associated with male infertility factors—a significantly confounding variable in correctly identifying high quality oocytes for fertilization in IVF. After applying LDC, deep learning models for binary classification of several kinds of segmented oocyte image inputs led to a significant uplift in performance for predicting oocyte competence, from 61.8 to 73.1% on average—a 18.4% increase.

The performance increase for oocyte classification was dependent on the segmentation style. Models trained on unsegmented images exhibited a total accuracy performance of 77.5%, greater than that of Intrazonal cavity (IZC) segmented images (71.7%), and that of *zona pellucida* segmented images (70.1%). These observations suggest that there could be a synergistic effect in the interplay between the IZC quality and the *zona pellucida* for evaluating oocyte competence, where the predictive power of each one alone is reduced. Historical literature supports the view of *zona pellucida* morphology as a significant indicator of oocyte quality^44,45^, and it is noted here that masking this component from the image reduces the overall predictive power. This may indicate value in ensembling or distilling models trained on segmented and unsegmented images.

These results indicate that automating detection of errors in datasets can be made scalable and robust, without human oversight that would violate data privacy. Given the growing cost of manual data verification and cleansing on increasingly Big Data around the world, this paper provides a pivotal framework for automated algorithms to detect data errors, for and beyond AI applications.

## Data Availability

The Human Activity Recognition **(HAR)** dataset is publicly available at https://www.kaggle.com/datasets/die9origephit/human-activity-recognition. The Patient Care dataset is also publicly available at https://www.kaggle.com/datasets/saurabhshahane/patient-treatment-classification. The Oocyte Image dataset generated during the current study is not publicly available due to ethical considerations and data privacy restrictions. Administrative permissions were required to access the Oocyte Image dataset, and were provided by M.D.V. The Oocyte Image dataset was anonymized before use.
