# Author Correction: Efficient automated error detection in medical data using deep-learning and label-clustering

**DOI:** 10.1038/s41598-023-49384-8

**Published:** 2023-12-19

**Authors:** T. V. Nguyen, S. M. Diakiw, M. D. VerMilyea, A. W. Dinsmore, M. Perugini, D. Perugini, J. M. M. Hall

**Affiliations:** 1 Presagen, Adelaide, SA 5000 Australia; 2 School of Computing and Information Technology, University of Wollongong, Wollongong, NSW 2522 Australia; 3 Ovation Fertility, Austin, TX 78731 USA; 4 Texas Fertility Center, Austin, TX 78731 USA; 5 California Fertility Partners, Los Angeles, CA 90025 USA; 6 Adelaide Medical School, The University of Adelaide, Adelaide, SA 5000 Australia; 7 Australian Research Council Centre of Excellence for Nanoscale BioPhotonics, Adelaide, SA 5005 Australia; 8 School of Physical Sciences, The University of Adelaide, Adelaide, SA 5005 Australia

Correction to: *Scientific Reports* 10.1038/s41598-023-45946-y, published online 09 November 2023

The Acknowledgements section in the original version of this Article was incomplete.

“The authors appreciate the collaboration and support of California Fertility Partners' embryologists (Claudia DeRomana, Michelle Kelly, Ahmad Abu Maizar, Melanie Nordbak, Pedro Toledo), and physicians (Drs. Kelley Baek, Korine Chung, Richard P. Marrs, Guy Ringler), in making data collection possible for this study.”

now reads:

“The authors appreciate the collaboration and support of California Fertility Partners’ embryologists (Claudia DeRomana, Michelle Kelly, Ahmad Abu Maizar, Melanie Nordbak, Pedro Toledo), physicians (Drs. Kelley Baek, Korine Chung, Richard P. Marrs, Guy Ringler) and their Lab Director (Mitchel Schiewe) in making data collection possible for this study.”

The original Article has been corrected.

